# Ionophore-Based Polymeric Sensors for Potentiometric Assay of the Anticancer Drug Gemcitabine in Pharmaceutical Formulation: A Comparative Study

**DOI:** 10.3390/molecules28227552

**Published:** 2023-11-12

**Authors:** Gamal A. E. Mostafa, Maha F. El-Tohamy, Essam A. Ali, Rashad Al-Salahi, Mohamed W. Attwa, Haitham AlRabiah

**Affiliations:** 1 Department of Pharmaceutical Chemistry, College of Pharmacy, King Saud University, Riyadh 11451, Saudi Arabiaralsalahi@ksu.edu.sa (R.A.-S.); mzeidan@ksu.edu.sa (M.W.A.);; 2 Department of Chemistry, College of Science, King Saud University, Riyadh 11495, Saudi Arabia; moraby@ksu.edu.sa

**Keywords:** gemcitabine, ionophore, host–guest, PVC sensors, potentiometry, quality control

## Abstract

Gemcitabine is a chemotherapeutic agent used to treat various malignancies, including breast and bladder cancer. In the current study, three innovative selective gemcitabine hydrochloride sensors are developed using 4-tert-butylcalix-[8]-arene (sensor 1), β-cyclodextrin (sensor 2), and γ-cyclodextrin (sensor 3) as ionophores. The three sensors were prepared by incorporating the ionophores with *o*-nitrophenyl octyl ether as plasticizer and potassium tetrakis(4-chlorophenyl) borate as ionic additive into a polyvinyl chloride polymer matrix. These sensors are considered environmentally friendly systems in the analytical research. The linear responses of gemcitabine hydrochloride were in the concentration range of 6.0 × 10^−6^ to 1.0 × 10^−2^ mol L^−1^ and 9.0 × 10^−6^ to 1.0 × 10^−2^ mol L^−1^ and 8.0 × 10^−6^ to 1.0 × 10^−2^ mol L^−1^ for sensors 1, 2, and 3, respectively. Over the pH range of 6–9, fast-Nernst slopes of 52 ± 0.6, 56 ± 0.3, and 55 ± 0.8 mV/decade were found in the same order with correlation regressions of 0.998, 0.999, and 0.998, respectively. The lower limits of detection for the prepared sensors were 2.5 × 10^−6^, 2.2 × 10^−6^, and 2.7 × 10^−6^ mol L^−1^. The sensors showed high selectivity and sensitivity for gemcitabine. Validation of the sensors was carried out in accordance with the requirements established by the IUPAC, while being inexpensive and easy to use in drug formulation. A statistical analysis of the methods in comparison with the official method showed that there was no significant difference in accuracy or precision between them. It was shown that the new sensors could selectively and accurately find gemcitabine hydrochloride in bulk powder, pharmaceutical formulations, and quality control tests. The ionophore-based sensor shows several advantages over conventional PVC membrane sensor sensors regrading the lower limit of detection, and higher selectivity towards the target ion.

## 1. Introduction

**Figure 1:**
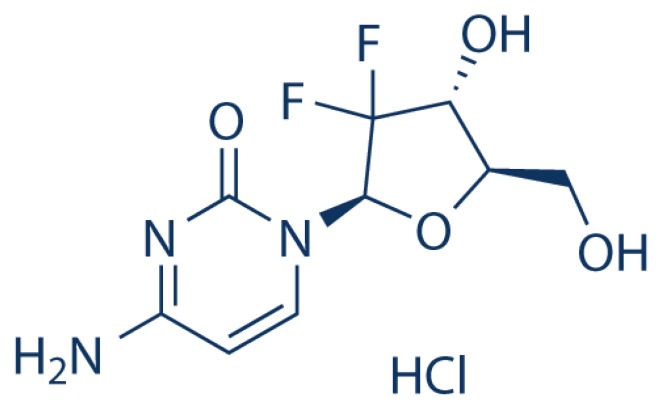
Chemical structure of gemcitabine hydrochloride.

Gemcitabine hydrochloride (GT) is a chemotherapeutic agent and a nucleoside analog. It was originally studied for its antiviral properties, but it is now used in cancer therapy for a variety of malignancies [1,2]. It is a cytidine analog in which the hydroxyl on the ribose is replaced by two fluorine atoms. As a prodrug, gemcitabine is converted into active metabolites that inhibit tumor development and cause cancer cells death by replacing nucleic acid building blocks during DNA elongation, stopping tumor growth, and promoting apoptosis in malignant cells [3]. It has a similar structure, metabolism, and mode of action to cytarabine, but provides a broader spectrum of antitumor activity [4]. The structure of gemcitabine hydrochloride is shown in Figure 1 [5].

Various analytical methods have been used to determine GT in its dosage forms or in biological fluids, including HPLC [6,7,8,9,10,11], HPLC-MS [12], LC-MS/MS [13], UV spectrophotometry [14,15,16], spectrofluorimetry [17], and voltammetry [18,19,20]. However, these methods usually require sample preparation and are time-consuming and costly. Potentiometric PVC membrane sensors are widely used for the analysis of pharmaceutical compounds because they are highly sensitive, simple, and easy to miniaturize, have a low price, and require a relatively short analysis time [21].

Chemical sensors are devices that can convert information about the chemical composition of a particular medium into a signal that can be easily detected. Usually this is an electrical signal (electromotive force, current, conductivity, capacitance), but it can also be an optical, acoustic, or other signal. Chemical sensors generally provide non-destructive, in-place analysis and in-line monitoring; they are energy and cost efficient, easy to operate and maintain, and require relatively simple and affordable ancillary equipment. Because of these advantages, chemical sensors are widely used in a variety of applications [22].

The interaction between ionophore and analyte in ion-selective electrodes improves the sensitivity, biocompatibility, and stability of the sensor response for analyte ions and lowers the detection limit [23]. Supramolecular chemistry is used to introduce cyclodextrin (CD) and calixarene derivatives as macrocycle molecules [24]. They act as ionophores in sensors used to analyze a variety of pharmaceutical compounds [25]. Noncovalent (H-bond, ionic, van der Waals, or hydrophobic) interactions are used to coordinate cationic, anionic, or neutral guests in discrete host molecules or supramolecular host assemblies [26]. While this notion sets useful limits in the well-established domain of organic hosts (such as crown ethers, calixarenes, and porphyrins), considerable thought is required in extending this concept to host assemblies composed of atoms other than carbon.

Cyclodextrins are known to accommodate a variety of organic, inorganic, and biological guest molecules in their hydrophobic cavity to form stable host–guest inclusion complexes or supramolecular nanostructures while providing high molecular selectivity and enantioselectivity [27,28]. For the determination of various active pharmaceutical ingredients such as ibuprofen using different commercial types of cyclodextrins [29], naproxen using different potentiometric sensors based on neutral, heptakis(2,3,6-tri-O-methyl), hepatakis (2,3,6-tri-O-benzoyl)-β-cyclodextrin, and anionic β-cyclodextrin [30], and the enantioselective high-performance potentiometric sensor based on a sulfated γ-cyclodextrin–carbon nanofiber composite for the determination of moxifloxacin were used [31].

The cyclic oligomers known as 4-tert-butylcalix-[8]-arenes (calixarenes) consist of phenolic units linked by alkylidene groups. They have a variety of selective properties in their structure, including cavity size, conformation, and substituents, which lead them to form typical host–guest complexes with a variety of substances. This allows them to be used in a number of different ion-selective membrane and electrode applications. Electrostatic contacts, hydrogen bonds, and van der Waals forces are some examples of non-covalent interactions that contribute to the formation of host–guest interactions, also known as inclusion complexes [32]. On the other hand, coordination contact between the contributing atom and the guest promotes the formation of the inclusion complex.

As far as we know, we used cyclodextrin and calixarene ionophore as sensing materials for the first time in the construction of PVC membrane sensors for gemcitabine. To the best of our knowledge, this was the first study to use cyclodextrin and calixarene ionophore as sensing materials in the construction of PVC membrane sensors for gemcitabine detection. In comparison to earlier research using GT-PT ion pairs, the current study has the benefit of being more sensitive and selective [5]. In comparison to previously published methods (10^−5^ M), the detection limits of the current study are more sensitive (10^−6^ M).

The goal of the current investigation is to determine GT employing KTpClPB as an ion additive, *o*-NPOE as a plasticizer, and PVC matrix using calixarene, β-CD, γ-CD, and calixarene as ionophores for the construction of the suggested sensors 1, 2, and 3, respectively. The accuracy and precision of the suggested sensors were investigated, and the designed sensors were applied for the determination of gemcitabine in bulk and injection solution. Investigations on the appropriateness and validity of potentiometric systems have been carried out in accordance with the analytical requirements and methodology that have been approved by IUPAC. The suggested sensors were examined, and the designed sensors were used to determine the presence of gemcitabine in injection fluid and bulk.

## 2. Results and Discussion

### 2.1. Mechanism of Sensing Membrane

**Scheme 1:**
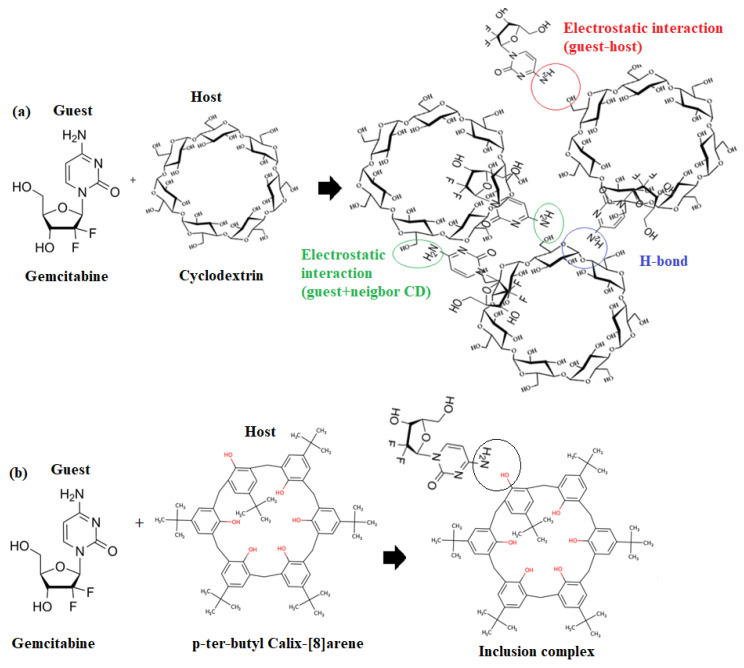
Host and guest inclusion of (**a**) gemcitabine-CD and (**b**) gemcitabine -4-ter-butylcaix [8] arene.

Ion-selective membrane sensors have a potential difference between the sample and membrane phase at the membrane junction. Here, the ions of interest are detected [33]. As shown in Scheme 1a, modified cyclodextrins are considered molecular receptors and cooperative binding with specific guest molecules has been mainly attributed to intermolecular interactions (hydrogen bonds, hydrophobic interactions, and van der Waals forces) that support cooperative binding processes between the host and guest molecules [34]. Although the size and geometry of the guest most influence the binding strength, the host–guest interactions can be influenced by changing the three hydroxyl groups on each glucose molecule. On the other hand, calixarenes are recognized as being capable of acting as selective ligands for a variety of ions by means of dipole–dipole interactions, as shown in Scheme 1b. On the other hand, GT are composed of donor atoms such as oxygen and nitrogen, which help to maintain the coordination relationship that exists between the host and the guest. Moreover, the positive charge of GT makes the process of coordination between the guest and the host easier to accomplish by generating a flexible inclusion complex reaction (Scheme 1).

### 2.2. Charactrization of Inclusion-Complexes 

#### 2.2.1. FT-IR Analysis of Inclusion-Complexes 

**Figure 2:**
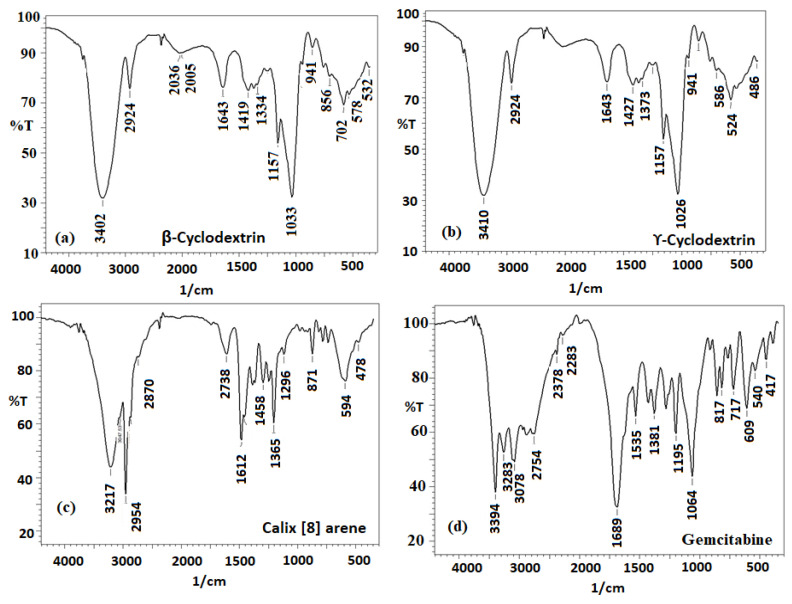
FT-IR spectra of (**a**) β cyclodextrin, (**b**) ϒ-cyclodextrin, (**c**) calix [8] arene, and (**d**) gemcitabine.

The synthesized solid inclusion complexes of GT- β-CD, GT-γ-CD, and GT_calixarene were then studied using a critical and decisive FT-IR spectral analysis. The FT-IR spectrum analysis clearly confirmed the non-covalent molecular interaction between the host and guest during inclusion [35]. Due to the altered electron density in their vicinity, the confinement phenomenon resulted in changes in peak intensity, shape, and location, as well as the removal of several peaks in the FT-IR spectra of both drug and host [36]. Figure 2a–d show the infrared spectra of-β-CD, γ-CD, calixarene and GT as well as their chemical bonds.

FT-IR spectra of both β-CD and ϒ-CD showed different absorption bands at 3402 and 3410 cm^−1^ (strong broad O-H stretching frequency), 2924 cm^−1^ (medium C-H stretching of the alkane), 2036 and 2005 cm^−1^ (weak -C-H bending of the alkane), 1643, 1427, and 1419 cm^−1^ (medium -C-H bending of alkane), 1373 and 1334 cm^−1^ (medium O-H bending of phenol), 1157, 1026, and 1033 cm^−1^ strong C-O stretching vibration. 941–702 cm^−1^ (strong C-H bending of trisubstituted phenol), and 578–524 cm^−1^ (strong C-halo compound), respectively (Figure 2a,b). The FT-IR spectrum of calix [8] arene showed the appearance of significant bands at 3217, 2954, 2870, 2738, 1612, 1458, 1365, 1296, 871, 594, and 478 cm^−1^ corresponding to the O-H stretching of the carboxylic acid, weak O-H stretching of alcohol, medium C-H stretching of alkane, medium C-H stretching of aldehyde, medium C=C stretching of conjugated alkene, medium C-H bending of alkane, strong C-O stretching of aromatic esters, C=C bending of alkene, or C-halide of halo compound, respectively (Figure 2c). On the other hand, the spectrum of gemcitabine showed significant bands at 3394 cm^−1^ (medium N-H stretching of amine salt), 3283 cm^−1^ (medium N-H stretching of aliphatic primary amine), 3078 cm^−1^ (strong O-H stretching of carboxylic acid), 2754 cm^−1^ (medium C-H stretching of aldehydes), 2283 cm^−1^ (strong O=C=O stretching of carbon dioxide), 1689 cm^−1^ (weak C-H bending of the aromatic compound), 1535 cm^−1^ (strong N-O stretching of the nitro compound), 1195 cm^−1^ (strong C-F stretching of fluorine compound), 1064 cm^−1^ (strong C-O stretching of an alkyl aryl ether), 817–609 cm^−1^ (Strong C=C bending of alkene), and 540–417 cm^−1^ (strong C-F of a halogen compound), respectively (Figure 2d). 

**Figure 3:**
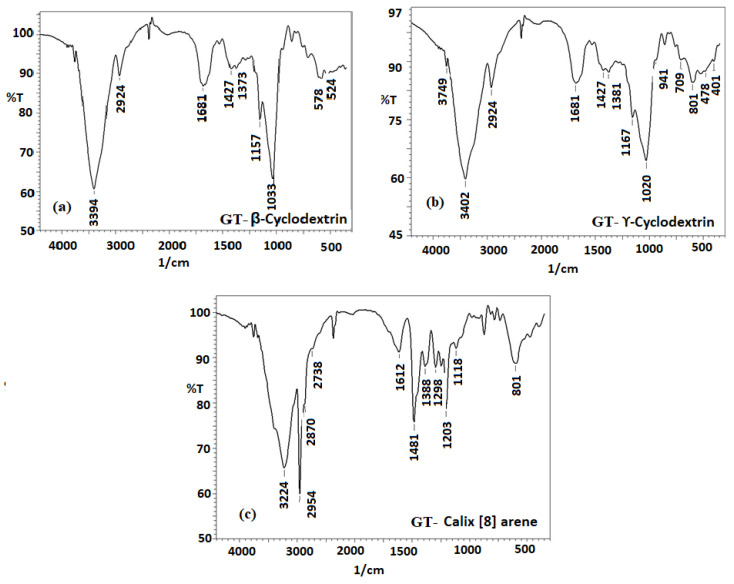
FT-IR spectra of (**a**) GT-β cyclodextrin, (**b**) GT-ϒ- Cyclodextrin, and (**c**) GT-calix [8] arene.

Sharp peaks at 3394 cm^−1^ (medium N-H stretching of aliphatic primary amine), 3402 cm^−1^ (medium N-H stretching of primary amine), and 3224 cm^−1^ (medium C-H stretching of the alkene) were observed in the spectra of (GT-β-CD), (GT- ϒ-CD), and GT-calix [8] arene, respectively (Figure 3a–c), whereas broad peaks corresponding to O-H and N-H were recorded for pure (β-CD), (ϒ-CD), calix [8] arene, and GT. The complete change in the nature of the peaks may be due to the involvement of—OH binding of both the drug and the hosts in the formation of the interaction complex. Moreover, the presence of a strong peak in the inclusion complexes clearly indicates that the free NH_2_ bond is not in contact with the host. Encapsulation occurred over the five-membered ring, leaving the six-membered ring holding the NH_2_ bond from GT relatively free. These results were further confirmed by the complete disappearance of the OH bending vibration of the host metal. The C-O stretching frequency of GT, which was observed at 1064 cm^−1^, shifted to 1033 and 1020 cm^−1^ for GT -β- CD), (GT—ϒ- CD inclusion complexes, respectively [37].

#### 2.2.2. NMR Analysis of Inclusion-Complexes 

**Figure 4:**
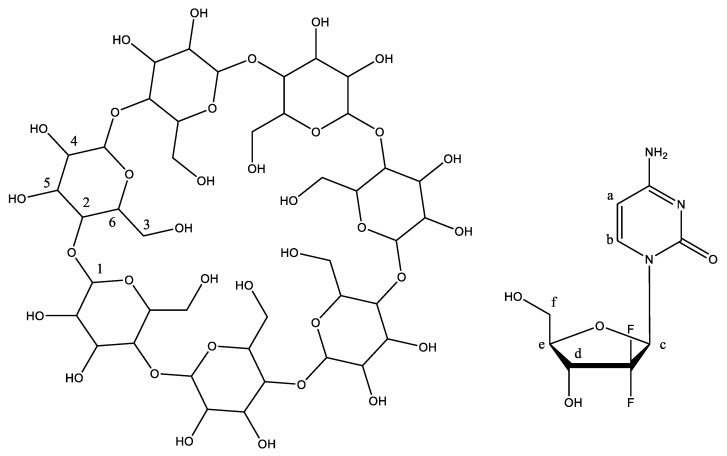
NMR confirms the formation of β-CD, gemcitabine, and β-CD/gemcitabine.

**Figure 5:**
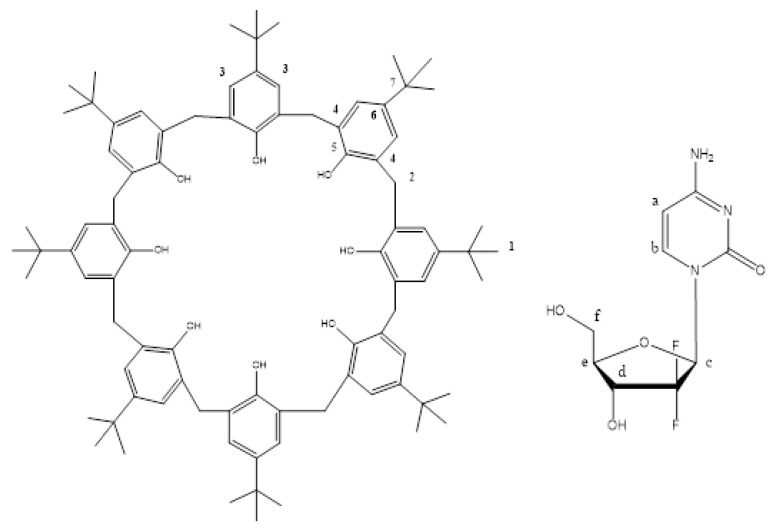
NMR analysis confirms the formation of gemcitabine -4-ter-butylcaix [8] arene.

The chemical shifts of both protons and carbons were obvious in the NMR data that was presented in Table 1, Table 2 and Table 3, and Figure 4 and Figure 5, which confirmed the formation of complexes 1–3.

### 2.3. Optimization Conditions 

#### 2.3.1. Ionic Additive 

The addition of KTpClPB as an ionic site to the membrane composition has a significant effect on membrane behavior by acting as an anionic exclusion factor, increasing membrane selectivity for the target analyte and reducing anionic interference, while also improving selectivity for the drug gemcitabine while neutralizing the charge generated between GT and the host [38,39]. Table S1 provides a summary of the results. At a concentration of 5 mg of the ion exchanger, the membrane composition was found to be optimal for sensors 1, 2, and 3. The addition of 5 mg (KpCTPB) improved the selectivity and sensitivity of the tested sensors. The host–guest interactions used as the basis for the design of these sensors show strong selectivity and virtually Nernstian response when used in the presence of ion exchange resins or lipophilic ions. These sensors exhibit high affinity for gemcitabine. The electrode response was measured after addition of 5 mg of the ion exchange resin. The optimal electrode response was 52, 56, and 65 millivolts per decade for sensors 1, 2, and 3, respectively.

#### 2.3.2. Effect of Plasticizers

Plasticizers play an important role in membrane properties by acting as fluidizers and facilitating complete dissolution of the membrane substance. Gemcitabine membrane sensors with three different ionophores (calixarene, β- CD and γ- CD) were evaluated using three different plasticizers (DOP, DBS and *o*-NPOE). The performance of the newly developed sensors using different plasticizers is shown in Table 4, which can be found here. The responses of the proposed sensors to different plasticizers (DOP, DBP and *o*-NPOE) were evaluated, and it was found that *o*-NPOE performed the best because it has a higher dielectric constant (*o*-NPOE, ε = 24) than DOP, ε = 5.1) and (DBS, ε = 4.5). As a consequence of the results described in the previous section, it was found that the use of *o*-NPOE gave the best potential response for sensors 1, 2, and 3. This can be attributed to the high dielectric constant of *o*-NPOE (ε = 24). Therefore, o-NPOE was selected for further investigations.

#### 2.3.3. Selectivity

The selectivity coefficients ($K_{A,\;B}^{pot}$) of GT sensors were determined according to the IUPAC guidelines using separate solution and mixed-solution methods [40,41]. Table 5 contains a listing of the selectivity values of the investigated sensors. Equation (1) was used to determine the selectivity based on the separate-solution method:

(1) $$\log K_{A,B}^{pot}=\frac{E_{B}-E_{A}}{S}+\left[1-\frac{Z_{A}}{Z_{B}}\right]\log a_{A}$$

whereas the selectivity values measured by match potential method were calculated using Equation (2):

(2) $$K_{A,B}^{pot}=\frac{\left({a‘}_{A}-a_{A}\right)}{a_{B}}$$

where log $K_{A,\;B}^{pot}$ represents log selectivity coefficient, *E*_A_ and *E*_B_ are the potentials of the proposed sensors for each GT and interfering species (1.0 × 10^−3^ mol L^−1^), resp., whereas Z_A_, Z_B_, ${a‘}_{A}$, and $a_{A}$ are the charges and activities of GT and interfering species, respectively. The results indicated that the three proposed sensors show good selectivity.

#### 2.3.4. Effect of pH

**Figure 6:**
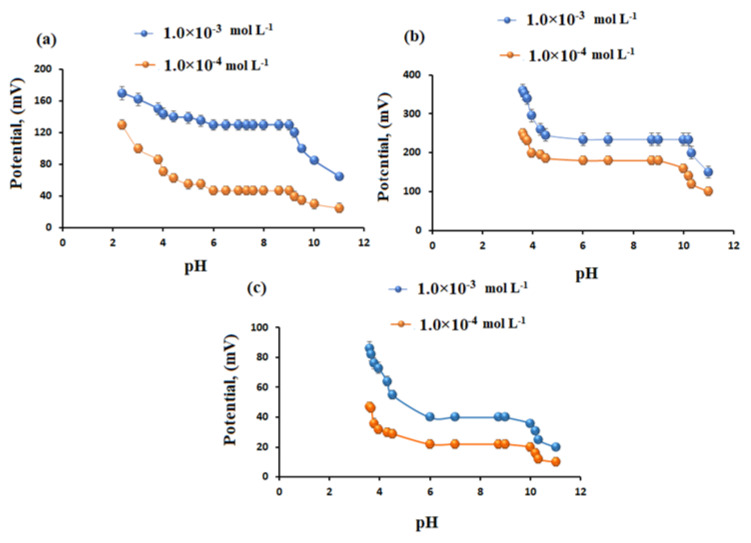
Effect of pH on the potential readings of (**a**) sensor 1 (calixarene), (**b**) sensor 2 (β−CD), and (**c**) sensor 3 (γ−CD) using 1.0 × 10^−3^ and 1.0 × 10^−4^ mol L^−1^ of GT.

The optimal pH for measuring GT in solution was determined utilizing sensors 1–3 at two GT concentrations (1.0 × 10^−3^ and 1.0 × 10^−4^ mol L^−1^) in pH range 2–12 (Figure 6). In the pH range 6–9, the responses of the sensors were almost constant, at 52 ± 0.6, 55 ± 0.8 and 56 ± 0.3 mV decade^−1^ for sensor 1, 2 and 3, resp. In acidic medium with a pH ≥ 4 the drug was unstable, and the potential readings gradually increased. whereas at pH values > 9 the drug dominantly exists as a free base, and potential decreased due to the formation of deprotonated species of GT [42].

#### 2.3.5. Response and Soaking Time

The response time of the GT sensors was approximately 20 s over the tested concentration of GT after 25 ± 1 s, and the potential reading was constant. The sensors exhibited a longevity exceeding two months, throughout which their slopes remained consistent, accompanied by a steady response time of 25 s. In order to investigate the effect that different soaking times have on the potential responses of sensors, they were submerged for 0.5, 1, 2, 3, 5, 6, and 12 h, respectively. It was observed that 3 h is adequate for conditioning new section of the membranes. The effect of immersion time on the electrode response was inserted in Table S2. 

### 2.4. Validation of the Method

The analytical characteristics of the investigated sensors 1, 2, and 3, using different ionophores and *o*-NPOE as plasticizers were optimized for GT determination. The validation of the investigated sensors was summarized as suggested by the IUPAC recommendation [40,41] (Table 6). The linearity of the calibration graph was calculated from Equation (3):

(3) $$E\left(mV\right)=S\;\log\;\left[GT\right]+intercept$$

**Figure 7:**
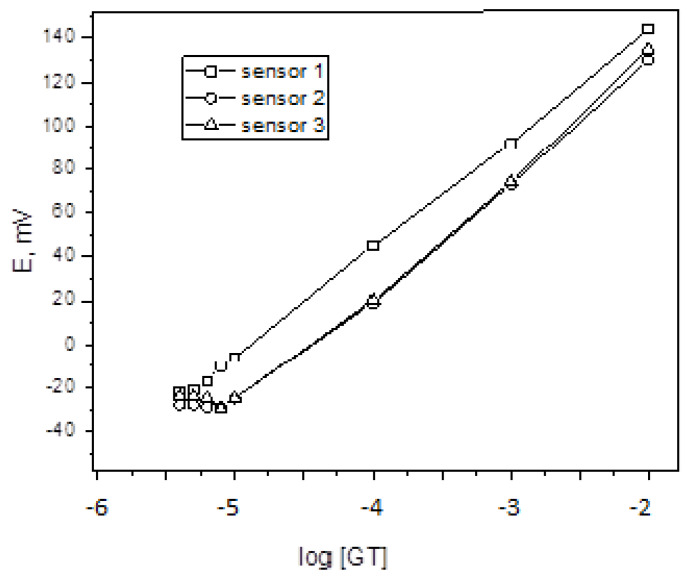
Calibration graph of the proposed sensors.

The measured potential of the sensor, denoted as E (mV), is influenced by various factors. One such factor is the slope, shown by the values 52 mV decade^−1^, 56 mV decade^−1^, and 55 mV decade^−1^ for sensors 1, 2, and 3, respectively. Additionally, the intercept values for the sensors are 179 mV, 299 mV, and 239 mV, respectively (Figure 7). The linearity of the response of sensors 1, 2 and 3 was maintained over 6.0 × 10^−6^–1.0 × 10^−2^, 9.0 × 10^−6^–1 × 10^−2^ and 8.0 × 10^−6^–1 × 10^−2^ mol L^−1^. The current study (calixarene, β-CD and γ-CD: 6.9 × 10^−6^, 9 × 10^−6^ and 8 × 10^−6^ mol L^−1^) is more selective and sensitive than prior work on GT-PT-ion-pairs employing carbon paste, coated wire, and PVC membrane, with detection limits of 6.5 × 10^−5^, 7.2 × 10^−5^, and 4.6 × 10^−5^, respectively [5].

*LOQ and LOD:* The lower limit of quantification (LOQ) was 6.0 × 10^−6^, 9.0 × 10^−6^, and 8.0 × 0^−6^ mol L^−1^, whereas *LOD* was 2.5 × 10^−6^, 2.2 × 10^−6^, and 2.7 × 10^−6^ mol L^−1^, for sensors 1, 2 and 3, respectively.

Accuracy and precision: The accuracy and precision were examined throughout the day and inter-day. The results are listed in Table 7. For sensors 1, 2 and 3, the accuracy was 97.8%, 98.3% and 97.9% for intra-day, and 97.6, 97.9 and 97.6% for inter-day. On the other hand, RSD was 2.3% for intra-day, whereas for inter-day it was 3.0%, 2.5%, and 2.9%, for sensors 1, 2 and 3, respectively. The results revealed the fair accuracy and precision of the method.

Ruggedness: Analyses of gemcitabine employing two separate analysts and two different instruments were used to test the robustness of the established potentiometric approaches. RSD was <3.1% using two different instruments and two analysts. The results revealed the fair accuracy and precision of the method.

### 2.5. Application 

The direct determination of 2.36–263.2 µg mL^−1^ GT (*n* = 5) in bulk form was carried out. An average recovery of 98.1%, 98.3% and 98.2% and precision of 2.3%, 2.2%, and 2.3% for sensors 1, 2 and 3, respectively, were evaluated (Table 8). GT was determined in an injection solution utilizing the studied sensors (Table 9). The results were compared to those that were acquired by the spectrophotometric method that had been reported [14]. Student’s t-test was used to assess the accuracy of both approaches, while the *F*-test was used to assess precision. The findings showed that the sensors displayed recoveries of 97.5 ± 2.5%, 98.5 ± 2.1%, and 98.0 ± 2.9%, and calculated (|t|_2_ are 1.3 and 1.5) and F-test (1.5 and 1.7) (*p* = 0.05 and *n* = 5), being lower than the tabular values, (|t|_2_ = 2.766) and F-test = 6.39) indicated that the suggested potentiometric methods are in good agreement with the literature method and each other in terms of accuracy and precision [43]. 

### 2.6. Stability and Repeatability

The most important limitations of solid selective sensors are the stability and repeatability of the fabricated sensors. The stability and repeatability of the proposed ionophore-based sensors have been studied from this point of view. The stability and repeatability of the fabricated sensors were performed by repeating the measurements five times using the pre-fabricated sensors over the GT concentration range 2.36–263.2 µg mL^−1^; the mean percent recoveries were found to be 98.33 ± 0.8%, 98.08 ± 0.7%, and 98.16 ± 0.6%. The obtained findings revealed good stability and repeatability of the designed potentiometric systems. 

### 2.7. Comparison of Ionophore-Based PVC Sensors with Reported Potentiometric Method

After a lot of research, only one potentiometric method was found for the determination of GT [5]. Ion-pair complexes of GT-tetraphenylborate are used as a detecting material in the method that was reported. Carbon paste sensors, coated wire sensors, and PVC sensors [5] are the three types of sensors that are constructed. The comparison of the investigated method with the reported method is listed in Table 10. In the present study, three sensitive ionophore-based sensors were developed for the determination of GT. The results show high selectivity and sensitivity for the determination of GT with a wide concentration range, a low detection limit, and good stability and repeatability.

## 3. Experimental

### 3.1. Instruments

All potentiometric measurements were made using an Orion pH/mV meter (model 330) from ThermoFisher Scientific,, Waltham, MA, USA, using GT sensors and Ag/AgCl reference electrode (model 90-02, Orion, ThermoFisher Scientific). Glass pH electrode (Orion 81-02) was used to measure pH. Deionized water was created using a Milli-Q plus purification water system (Millipore; Billerica, MA, USA). Chemaxon Windows 10.0 software has been utilized.

### 3.2. Materials

The reagents utilized in the experiment were of analytical purity grade. During the course of the experiment, double-distilled water from Millipore (Millipore, USA) was utilized. Sigma-Aldrich (Merck KGaA, Germany) supplied polyvinyl chloride (PVC) powder, high-molecular mass, dioctyl phthalate (DOP, 99.5%), dibutyl sebacate (DBS, 97.0%), o-nitrophenyl octyl ether (*o*-NPOE, 99.0%), and tetrahydrofuran (THF, >99%). Potassium tetrakis (4-chlorophenyl) borate (KTpClPB, ≥98.0%), β-CD (M.Wt. 1134.987, density: 1.46 g/cm^3^) and γ-CD (98.0%) (mol wt 1297.128 and density 1.41 g/cm^3^) and 4-*tert*-butylcalix [8] arene (calixarene) (97.0%) (mol wt 1297.8 and density 1.095 g/cm^3^) were obtained from BDH (UK). Gemcitabine hydrochloride (GT) of purity 98% was obtained from Med Chem Express (USA). The gemcitabine ampoules, namely the GEMZAR^®^ 200 mg infusion solution, was procured at the nearby pharmacy. A suitable amount of GT was dissolved in double-distilled water to create a stock solution with a concentration of 0.01 mol L^−1^. This stock solution was then diluted in a serial manner using water to produce five working solutions with concentrations ranging from 1.0 × 10^−6^–1.0 × 10^−2^ mol L^−1^ of GT. This was carried out using a sodium acetate and acetic acid buffer with a pH of 7. For pH measurement 0.05 mol L^−1^ HCl and NaOH were used.

### 3.3. General Strategy of this Study 

**Scheme 2:**
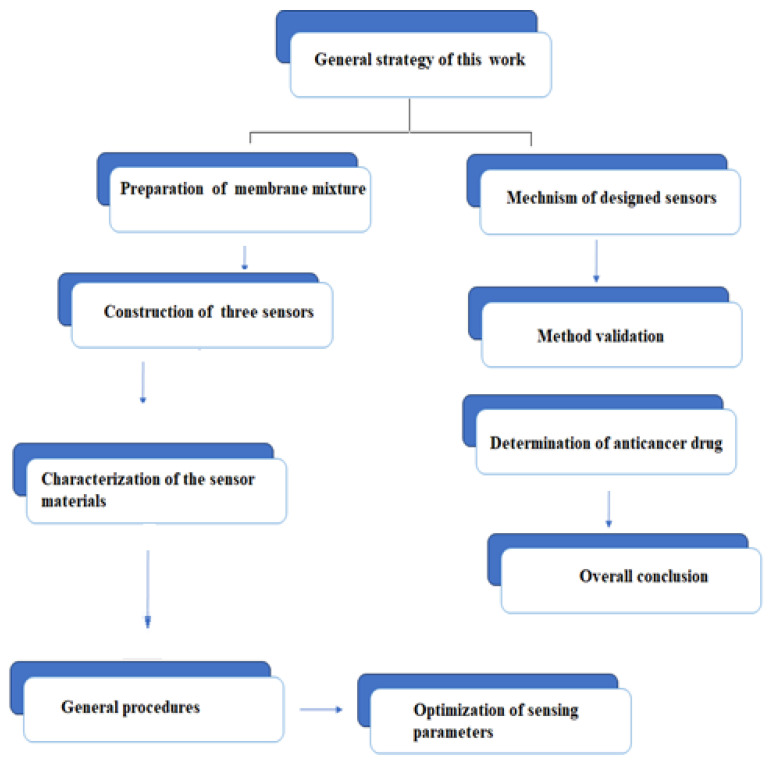
Schematic diagram that describes the general strategy of this study.

The following diagram describes the general strategy of the present work:

In brief. This study deals with the fabrication of three sensors with different ionophores. The preparation of the membrane sensors was conducted subsequent to the optimization of the components employed for the composition of the membranes. FT-IR and NMR, on the other hand, were used to study the inclusion complexes formed between GT and ionophores (β-CD, γ-Cd, and calixarene). Then, the mechanism of the fabricated sensors was explained. The proposed sensor systems were validated. Finally, the developed sensors were applied for the determination of GT, and the overall conclusion was summarized at the end of this study (Scheme 2).

### 3.4. Preparation of GT- β, γ-CD and GT-Calix [8] arene Inclusions

The kneading process was used to create the complexes [44]. The hydrochloride salt of GT and the corresponding β, γ-CD and Calix [8] arene inclusions were weighed in a 1:1 molar ratio. To make a paste, cyclodextrin was impregnated with a little amount of deionized water. GT was then added to the mixture and kneaded for roughly 5 min. The resultant combination was then dried at 40 °C for 24 h, yielding a white powder product that proved to be effective GT-CD or GT-calix [8] arene complex. The physical combination was created by carefully combining an appropriate quantity of GT with a specific CD or calix [8] arene in a molar ratio of 1:1.

### 3.5. Preparation of the GT-PVC Sensors

For the preparation of GT-PVC sensors, 25.0 mg of calixarene, β- or γ-cyclodextrin, and 5.0 mg KTpClPB were mixed with 190 mg PVC powder and 0.35 mL plasticizer (DOP, DBS, or o-NPOE) in a glass Petri dish (5 cm diameter). The mixture was thoroughly dissolved in an appropriate amount of THF. The detector membranes were prepared by incubating the Petri dish overnight as previously reported [45], and then cutting the membranes and bonding them to the sensor body. An internal reference electrode (Ag/AgCl) was used to construct the electrode, and an equal volume of equimolar 1.0 × 10^−2^ mol L^−1^ GT:KCl *v/v* reference solution was poured into the sensor body. The prepared sensors were conditioned by immersing each in a dilute GT solution and then stored in the same solution for further use. Potentiometric measurements were performed at a temperature of 25 ± 1 °C using the standard cell setup with the external reference sensor Ag/AgCl.

### 3.6. Characterization of Inclusion-Complexes

The solid inclusion complexes of GT with ionophores (β-CD, g-CD, and calixarene) were then examined using a critical and decisive FT-IR spectroscopic approach. The FT-IR spectrum analysis clearly validated the noncovalent molecular interaction between the host and the guest during inclusion [35]. Because of the change in electron density in their vicinity, the inclusion phenomenon caused changes in peak intensity, shape, and location, as well as the removal of several peaks in the FT-IR spectra of both the drug and the host [36]. Also, the inclusion complexes have been confirmed using NMR to determine their 1H and 13C. 

### 3.7. General Procedure and Calibration Graphs 

The calibration of GT membrane sensors by putting each sensor in, combined with a reference electrode (Ag/AgCl), in 30 mL of a GT (1 × 10^−2^ to 1 × 10^−6^ mol L^−1^) solution at pH 7 with continuous stirring. For each GT concentration, the potential (E, mV) was measured after reaching a steady state. The potential was then plotted against − −log [GT] to generate a calibration curve. The obtained regression equation was used to determine the unknown concentration.

### 3.8. Effect of ion Additives

The effect of the ion exchanger concentration on the electrode response was examined. The characteristics performance of the suggested sensors was tested using different concentration of ion exchanger hanger ranging from 0 mg to 7 mg in the presence of 25 mg of ionophore.

### 3.9. Effect of Immersion Time

The effect of immersion time on the electrode response was tested using different soaking times from 0.25 to 120 h. 

### 3.10. Determination of Gemcitabine in Ampoules 

An appropriate mass of GT solution (29.6 mg) was then transferred from the ampoules into a 100-mL beaker, diluted with water, and sonicated for about five minutes, the solution was then transferred into a 100-mL volumetric flask and completed to the mark with water. One mL of the prepared solution was inserted into a measuring cell containing acetate buffer with continuous stirring, and the potential was measured. The unknown concentration was calculated from the calibration graph.

### 3.11. Validation of the Method 

Logarithmic is the relationship that exists between the average potential and the concentration that the sensors have detected, in accordance with the Nernstian Equation (4):

*E* = *E*_0_ + *S* log [concentration] (4)

where E represents the electrode potential, E_0_ stands for the standard electrode potential, and S represents the slope of the potential gradient. The validation was carried out in compliance with the conditions that were set forth by IUPAC [40]. LOD, is defined by IUPAC as the concentration of GT corresponding to the inter section of the extrapolated linear segment of the calibration graph. LOQ = 3.3 LOD. 

*Accuracy and Precision*: To find out how accurate the GT assay was, a known amount of GT was tested at five different concentration levels, ranging from 2.36 to 263.2 µg mL^−1^, using sensors that were constructed. Each sample was measured five times, and the precision of the designed potentiometric systems was estimated as the mean percent recovery. Intermediate precision, on the other hand, was evaluated using the intra- and inter-day method by performing the analysis during the day and for three consecutive days. The precision of the potentiometric systems was expressed as %RSD.

## 4. Conclusions

The current study suggests the fabrication of three sensors based on different ionophores sensing materials, including calixarene, β-CD and γ-CD as ionophores for the determination of gemcitabine. The optimal pH range was 6–9, indicating a fair efficiency with quick response times of 25 s. Calixarene has a wider calibration range than γ-CD and β-CD, with lower limits of detection of 6.0 × 10^−6^, 8.0 × 10^−6^ and 9.0 × 10^−6^, respectively. The detection of GT utilizing the described methods demonstrated remarkable accuracy and precision. The suggested sensors have a high degree of precision and accuracy when compared to the spectrophotometric method for determining GT in bulk and injection solutions. As a result, they are expected to be applicable for routine GT analysis in quality control laboratories. 

## Figures and Tables

**Table 1:** The ^1^H and ^13^C of β-CD, gemcitabine, and β-CD/gemcitabine.

^1^H	ppm (DMSO)			^13^C	ppm (DMSO)		
	δ β-CD	δ Gemcitabine	δ β-CD/Gemcitabine Complex		δ β-CD	δ Gemcitabine	δ β-CD/Gemcitabine Complex
H1	5.7		5.65	C1	102.25		102.20
H2	3.33		3.32	C2	82.15		81.00
H3	3.53		3.79	C3	60.30		60.37
H4	3.59		3.91	C4	72.52		69.56
H5	3.60		3.46	C5	72.86		72.88
H6	4.53		4.84	C6	73.51		73.54
Ha		6.09	6.15	Ca		95.77	95.53
Hb		8.89	8.08	Cb		144.05	144.19
Hc		6.24	6.41	Cc		96.30	90.11
Hd		3.80	3.60	Cd		81.80	81.87
He		4.21	4.19	Ce		83.98	84.39
Hf		3.66	3.63	Cf		59.13	59.25

**Table 2:** The ^1^H and ^13^C of ϒ-CD, gemcitabine, and ϒ-CD/gemcitabine.

^1^H	ppm (DMSO)			^13^C	ppm (DMSO)		
	δ ϒ-CD	δ Gemcitabine	δ Gemcitabine/ϒ-CD Complex		δ ϒ-CD	δ Gemcitabine	δ Gemcitabine/ϒ-CD Complex
H1	5.73		5.70	C1	102.12		102.14
H2	3.31		3.31	C2	81.38		81.39
H3	3.52		3.81	C3	60.43		60.42
H4	3.67		3.62	C4	72.63		72.65
H5	3.65		3.60	C5	73.04		73.05
H6	4.40		4.89	C6	73.33		73.37
Ha		6.09	6.18	Ca		95.77	95.23
Hb		8.89	8.09	Cb		144.05	144.20
Hc		6.24	6.34	Cc		96.30	95.22
Hd		3.80	3.92	Cd		81.80	81.71
He		4.21	4.21	Ce		83.98	83.99
Hf		3.66	3.64	Cf		59.13	59.20

**Table 3:** The ^1^H and ^13^C of 4-ter-butylcaix [8] arene, gemcitabine, and gemcitabine-4-ter-butylcaix [8] arene.

^1^H	ppm (DMSO)			^13^C	ppm (DMSO)		
	δ 4-ter-butylcaix [8] arene	δ Gemcitabine	δ Gemcitabine-4-ter-butylcaix [8] arene Complex		δ 4-ter-butylcaix [8] arene	δ Gemcitabine	δ Gemcitabine-4-ter-butylcaix [8] arene Complex
H1	1.29		1.32	C1	31.48		31.44
H2	2.30		2.36	C2	30.96		30.84
H3	7.23		7.18	C3	125.54		125.58
				C4	128.72		128.51
				C5	146.68		146..61
				C6	144.55		144.41
				C7	34.03		33.99
Ha		6.09	6.23	Ca		95.77	95.20
Hb		8.89	8.53	Cb		144.05	144.11
Hc		6.24	6.30	Cc		96.30	93.00
Hd		3.80	3.82	Cd		81.80	81.88
He		4.21	4.16	Ce		83.98	83.89
Hf		3.66	3.60	Cf		59.13	59.24

**Table 4:** The influence of plasticizer on the newly developed gemcitabine sensors.

Plasticizer	DOP	DBS	*o*-NPOE
Sensor 1	4-*tert*-butylcalix [8] arene		
Slope, mV/decade	50	49	51
Response time, sec	25	30	25
Calibration range, mol L^−1^	6.0 × 10^−6^–1.0 × 10^−2^	9.0 × 10^−5^–1.0 × 10^−2^	6.0 × 10^−6^–1.0 × 10^−2^
Sensor 2	β-cyclodextrin		
Slope, mV/decade	55	51	56
Response time, sec	20	25	20
Calibration range, mol L^−1^	9.0 × 10^−6^–1.0 × 10^−2^	1.0 × 10^−5^–1.0 × 10^−2^	9.0 × 10^−6^–1.0 × 10^−2^
Sensor 3	γ-cyclodextrin		
Slope, mV/decade	55	53	55
Response time, sec	25	30	20
Calibration range, mol L^−1^	6.0 × 10^−6^–1.0 × 10^−2^	1.0 × 10^−5^–1.0 × 10^−2^	8.0 × 10^−6^–1.0 × 10^−2^

DOP: Dioctylphathalate; DBS: dibutyl sebacate; and *o*-NPOE: *o*-nitrophenyl octyl ether.

**Table 5:** Selectivity coefficients of some interfering ions, using the proposed sensors.

Interferent	$K_{A,B}^{pot}$ Sensor 1	$K_{A,B}^{pot}$ Sensor 2	$K_{A,B}^{pot}$ Sensor 3
Na^+^	1.1 × 10^−3^	1.5 × 10^−3^	1.2 × 10^−3^
K^+^	1.2 × 10^−2^	1.2 × 10^−3^	1.3 × 10^−3^
Ca^2+^	1.1 × 10^−2^	1.3 × 10^−3^	1.2 × 10^−3^
Fe^2+^	1.2 × 10^−2^	1.4 × 10^−3^	1.7 × 10^−3^
Magnesium Stearate	1.2 × 10^−3^	1.2 × 10^−3^	1.2 × 10^−3^
Acetate	1.3 × 10^−3^	1.1 × 10^−3^	1.3 × 10^−3^
Phosphate	1.3 × 10^−3^	1.3 × 10^−3^	1.2 × 10^−3^
Benzoate	1.3 × 10^−3^	1.2 × 10^−3^	1.3 × 10^−3^
Caffeine	1.1 × 10^−3^	1.3 × 10^−3^	1.2 × 10^−4^
Citrate	1.2 × 10^−3^	1.5 × 10^−3^	1.2 × 10^−3^
Glucose *	1.4 × 10^−3^	1.5 × 10^−3^	1.7 × 10^−3^
Lactose monohydrate *	1.4 × 10^−3^	1.5 × 10^−3^	1.6 × 10^−3^
Starch *	1.1 × 10^−3^	1.4 × 10^−3^	1.7 × 10^−3^
Microcrystalline cellulose *	1.4 × 10^−3^	1.3 × 10^−3^	1.6 × 10^−3^

* Match potential method. Concentration of analyte and interferent are 1.0 × 10^−3^ M, unit of selectivity is absolute value.

**Table 6:** Analytical characteristics of the suggested gemcitabine sensors.

Parameter	Sensor 1 Calixarene	Sensor 2 β-CD	Sensor 3 γ-CD
Slope, (mV decade^−1^)	52.0 ± 0.6	56.0 ± 0.3	55.0 ± 0.8
Intercept, mV	179.0 ± 0.5	299.0 ± 0.5	239.0 ± 0.5
Correlation Coefficient, (r^2^)	0.998	0.999	0.998
Calibration, range mol L^−1^	6.0 × 10^−6^–1 × 10^−2^	9.0 × 10^−6^–1.0 × 10^−2^	8.0 × 10^−6^–1 × 10^−2^
LOQ, mol L^−1^	6.0 × 10^−6^	9.0 × 10^−6^	8.0 × 10^−6^
LOD, mol L^−1^	2.5 × 10^−6^	2.2 × 10^−6^	2.7 × 10^−6^
Response time of 1.0 × 10^−3^ mol L^−1^, s	25 ± 0.5	25 ± 0.5	25 ± 0.5
Working pH range	6–9	6–9	6–9

*n* = number of repetitions: 5.

**Table 7:** The accuracy and precision data obtained from the determination of GT using the three designed sensors using calixarene, β, and γ-CD sensors.

Concentration (µg mL^−1^)	Within-Day		
	Sensor 1	Sensor 2	Sensor 3
	Recovery, % ± RSD, %	Recovery, % ± RSD, %	Recovery, % ± RSD, %
2.36	97.5 ± 2.7	97.5 ± 2.8	97.5 ± 2.8
2.63	97.5 ± 2.7	97.5 ± 2.8	97.5 ± 2.8
13.15	98.0 ± 2.7	97.5 ± 2.7	97.5 ± 2.6
26.3	98.5 ± 2.0	98.0 ± 1.9	98.0 ± 1.9
131.6	99.0 ± 1.8	98.0 ± 1.8	98.0 ± 1.8
263.2	99.0 ± 1.8	99.0 ± 1.8	99.0 ± 1.7
Different days			
2.36	97.5 ± 2.9	97.5 ± 2.8	97.5 ± 2.9
2.63	97.5 ± 2.7	97.5 ± 2.8	97.5 ± 2.8
13.15	97.6 ± 2.8	97.5 ± 2.7	97.5 ± 2.6
26.3	98.0 ± 2.6	97.5 ± 2.5	97.5 ± 2.5
131.6	98.5 ± 2.3	98.0 ± 2.5	98.0 ± 2.2
263.2	98.5 ± 2.3	98.0 ± 2.3	98.0 ± 2.2

Average of 5 measurements ± RSD. RSD%, Relative standard deviation %.

**Table 8:** Direct determinations of gemcitabine in bulk form using PVC membrane sensors.

Tested Concentration (µg mL^−1^)	Sensor 1	Sensor 2	Sensor 3
	Recovery ± RSD	Recovery ± RSD	Recovery ± RSD
2.36	97.5 ± 2.8	97.0 ± 2.8	97.5 ± 2.7
2.63	97.5 ± 2.6	97.5 ± 2.5	97.5 ± 2.5
13.15	98.0 ± 2.4	98.0 ± 2.4	98.0 ± 2.3
26.3	98.5 ± 2.1	98.5 ± 2.0	98.5 ± 2.0
131.6	99.5 ± 2.0	98.5 ± 2.0	98.5 ± 2.0
263.2	99.0 ± 1.8	99.0 ± 2.0	99.0 ± 1.8

Average of five measurements ± RSD.

**Table 9:** Analysis of gemcitabine in injection solution using PVC membrane sensors.

Preparation	Sensor 1 R% ± RSD%	Sensor 2 R% ± RSD%	Sensor 3 R% ± RSD%	Reported Method [5] R% ± RSD%
1000 mg	97.5 ± 2.5	98.5 ± 2.1	98.0 ± 2.9	98.5 ± 2.8
*t*-test	0.17	0.11	0.13	
F test	1.6	1.2	1.15	

Average of 5 measurements: R, recovery % and RSD: relative standard deviation%. Tablet value of *t*-test (2.776) and F-test (6.39) (*p* = 0.05 and *n* = 5).

**Table 10:** The data obtained from the comparison of the published potentiometric methods and the current study.

Potentiometric Method	Sensing Material	Linear Range of Detection	LOD	Ref.
Carbon paste, coated wire and PVC sensors	Sodium tetraphenyl borate	1.0 × 10^−5^–1.0 × 10^−2^ M	6.50 × 10^−5^ M 7.20 × 10^−5^ M 4.60 × 10^−5^ M	[5]
Ionophore-based PVC	4-tert-butylcalix-[8]-arene (sensor 1), β-cyclodextrin (sensor 2), and γ-cyclodextrin (sensor 3)	6.0 × 10^−6^–1 × 10^−2^ M 9.0 × 10^−6^–1 × 10^−2^ M 8.0 × 10^−6^–1 × 10^−2^ M	2.5 × 10^−6^ M 2.2 × 10^−6^ M 2.7 × 10^−6^ M	Present study

## Data Availability

All data in the manuscript are available from all authors.

## Supplementary Materials

The following supporting information can be downloaded at: https://www.mdpi.com/article/10.3390/molecules28227552/s1, Table S1: Effect of ion-exchanger on the electrode response; Table S2: Effect of immersion time on the electrode response of 1 × 10^−4^ M of GT.

## Acronyms, Abbreviations, Symbols

β-CD—β-cyclodextrin; DBS—dibutyl sebacate; DOP—dioctyl phthalate; γ-CD—γ-cyclodextrin; calixarene—4-*tert*-btylcalix [8] arene; GT—gemcitabine hydrochloride; KTpClPB—potassium tetrakis (4-chlorophenyl) borate; *o*-NPOE—*o*-nitrophenyl octyl ether; PVC—polyvinyl chloride; THF—tetrahydrofuran.
