# Supplementary material for: Ionophore-Based Polymeric Sensors for Potentiometric Assay of the Anticancer Drug Gemcitabine in Pharmaceutical Formulation: A Comparative Study
Source: Molecules. 2023 Nov 12;28(22):7552. doi: 10.3390/molecules28227552 (PMC10673180; doi:10.3390/molecules28227552)
Supplement: Supplementary file 1 [file molecules-28-07552-s001.zip › molecules-2670503-supplementary.pdf]

Table S1. Effect of ion-exchanger on the electrode response.

**Ion-exchange, mg**	**Sensor 1**	**Sensor 2**	**Sensor 3**
	**Slope** ± SD		
0	40 ± 0.4	43 ± 0.5	42 ± 0.5
2	48 ± 0.4	53 ± 0.5	52 ± 0.5
3	52 ± 0.5	55 ± 0.4	54 ± 0.4
5	52 ± 0.3	56 ± 0.4	55 ± 0.3
7	52 ± 0.3	56 ± 0.3	55 ± 0.3

*slope ± SD (n=3)

Table S2. Effect of immersion time on the electrode response of $1x10^{-4}$M of GT

Soaking time, min	Sensor 1	Sensor 2	Sensor 3
0.25	20	10	11
0.5	25	15	15
1.5	35	20	20
2	40	20	20
2.5	40	20	20
3	40	20	20
24	40	20	20
48	40	20	20
72	40	20	20
96	40	20	20
120	40	20	20
